# Posterior neo-annular reconstruction: An alternative approach to mitral valve replacement for caseous mitral annular calcification

**DOI:** 10.1016/j.xjtc.2026.102558

**Published:** 2026-07-27

**Authors:** Nika Samadzadeh Tabrizi, Shawn Shah, Gösta B. Pettersson, Marc Gillinov, Haytham Elgharably, Bo Xu, Serge Harb, Shinya Unai

**Affiliations:** a Department of Thoracic and Cardiovascular Surgery, Cleveland Clinic, Cleveland, Ohio; b Division of Cardiothoracic and Transplantation Surgery, Department of Surgery, University of Southern Florida, Tampa, Fla; c Department of Cardiovascular Medicine, Cleveland Clinic, Cleveland, Ohio

**Figure:**
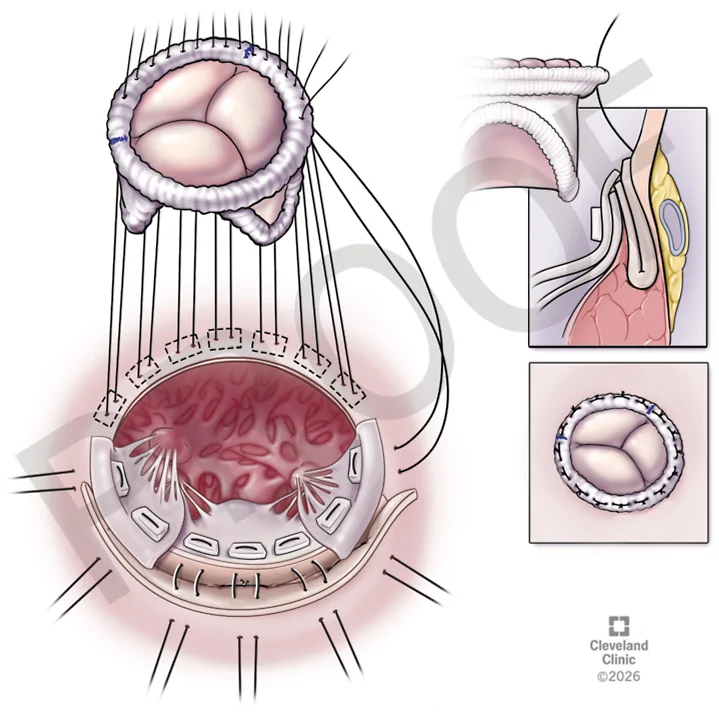
Cavity obliteration with pericardium and annular reconstruction with native leaflets.

**Central Message**

In CMAC, the cavity is evacuated and obliterated using autologous pericardium, and a robust mitral annulus is reconstructed and supported with preserved leaflets.

Caseous mitral annular calcification (CMAC) is rare, accounting for 0.5 to 1% patients with mitral annular calcification.^1^ Although the mechanism remains unknown, CMAC results from liquefaction and caseation within the calcified annulus, characterized by an annular mass filled with amorphous toothpaste-like material (Figure 1).^1^^,^^2^ Although often clinically indolent, surgical intervention is required in cases of severe mitral valve dysfunction, embolic phenomena, or progressive mass enlargement.

**Figure 1:**
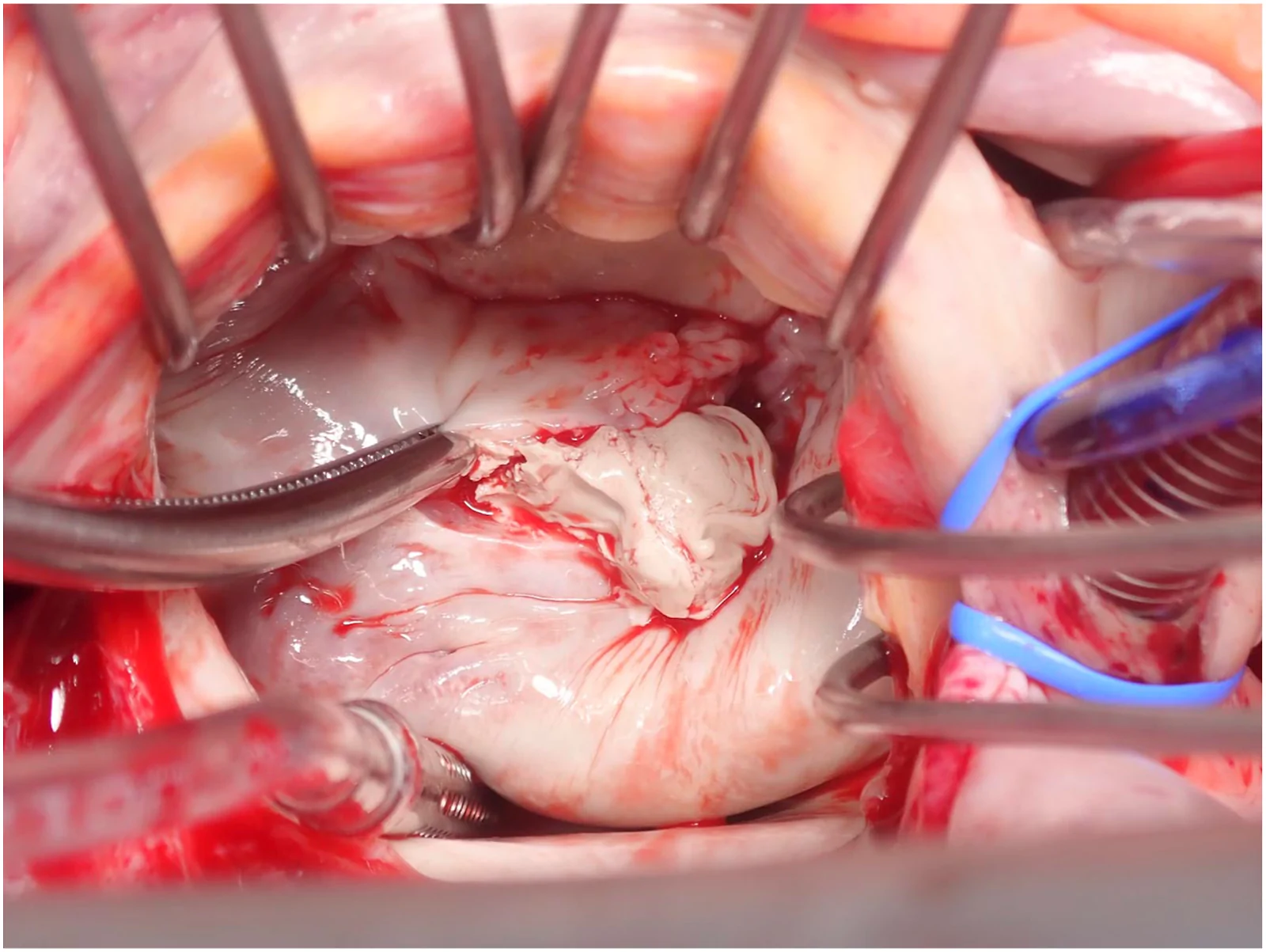
Incision of the mass along the posterior annulus revealed a large amount of caseous material.

Current literature offers limited guidance regarding optimal surgical management, particularly when evacuation of the caseous mass results in a large cavity with thin walls. We present our approach to mitral valve replacement in a 72-year-old woman presenting with progressive heart failure due to mitral regurgitation and CMAC measuring 3.5 × 2.2 cm (Figure 1, Video 1). Caseous material was completely evacuated, some denser mitral annular calcification was debrided, and the resulting cavity was obliterated with autologous pericardium. Valve sutures were placed through the robust reconstructed annulus, maximizing the use of residual leaflet tissue to buttress the posterior annulus and the thinned ventricular wall.

Institutional Review Board or equivalent ethics committee of Cleveland Clinic did not approve this study, and the need for patient written consent was waived for this publication because patient information was anonymized to ensure confidentiality.

## Surgical Principles for the Management of Caseous Mitral Annular Calcification

Traditional surgical approaches require aggressive debridement and patch exclusion followed by mitral valve replacement,^2^^,^^3^ whereas contemporary reports advocate for a more conservative approach.^4^^,^^5^ Traditional approaches have been associated with left ventricular rupture or perforation, atrioventricular groove disruption, fistula formation, and injury to the circumflex artery, potentially related to aggressive annular debridement.^1^^,^^3^^,^^4^ Conservative debridement may leave caseous material behind, provide insufficient support for a friable cavity wall, and allow embolization of residual debris.^4^^,^^5^ Suture placement through necrotic tissue and calcium also increases the risk of paravalvular leak or dehiscence.^3^ Debridement should prioritize complete evacuation of amorphous material while maximally preserving leaflet and subvalvular apparatus.

After evacuation, obliteration of the large cavity is equally essential. When CMAC extends into the atrioventricular groove, patch closure^4^ of the defect or direct closure using concentric spiral sutures^2^ has been described. Patch exclusion without cavity obliteration has been associated with pseudoaneurysm formation, recurrence, and embolization.^1^^,^^3^^,^^4^ Although technically challenging, direct suture obliteration of the cavity reduces residual dead space and thereby minimizes this risk.^2^^,^^5^ Our approach fills the cavity with autologous pericardium tacked to its base and incorporates residual native leaflet tissue for annular reconstruction, thereby obliterating the cavity and creating a durable scaffold for valve (Figure 2).

**Figure 2:**
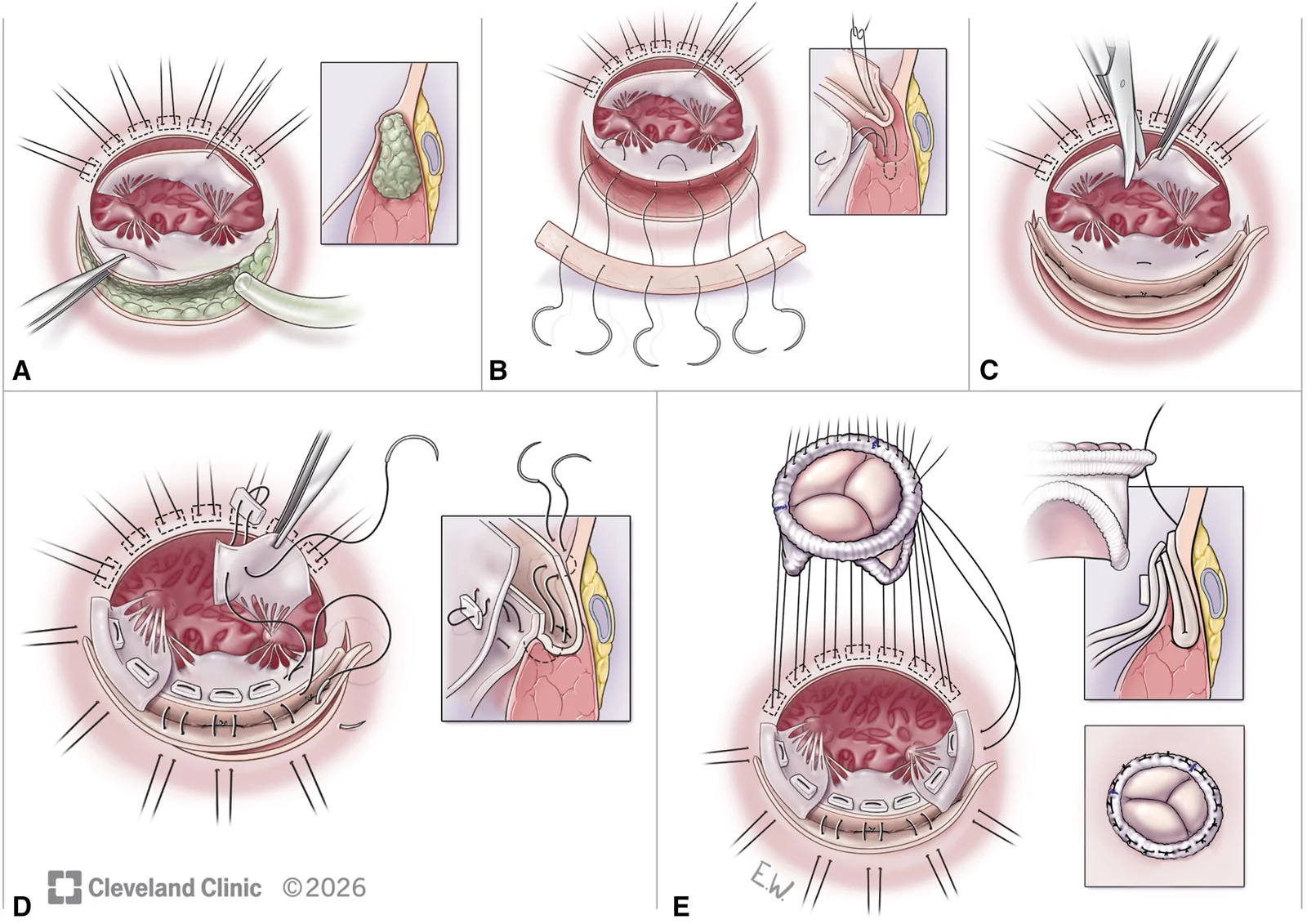
Mitral valve replacement for CMAC. A, Incision at the posterior annulus with evacuation of the caseous cavity, followed by incision at the anterior annulus. Noneverting interrupted pledgeted mattress sutures are placed along the anterior annulus, spanning trigone to trigone. B, To obliterate the cavity, native posterior leaflet and autologous pericardium are tacked into the base of the cavity. C, Division of the anterior leaflet. D, Noneverting interrupted pledgeted mattress sutures for the posterior annulus are passed through the native anterior leaflet and neo-posterior annulus, created by the native posterior leaflet and autologous pericardium. E, Sutures are passed through the sewing cuff of the valve. The valve is lowered into place, and valve sutures are tied.

Mitral valve repair may be considered in select patients with localized disease and preserved leaflets and subvalvular apparatus.^5^ However, most patients with CMAC and an indication for surgical intervention require mitral valve replacement. Evacuation of the caseous material from the cavity should not be compromised in pursuit of repair.

## Conclusions

Cavity evacuation and obliteration with autologous pericardium, together with preservation of leaflets and subvalvular apparatus, provide a well-supported mitral annulus for valve replacement in patients with CMAC.

## Conflict of Interest Statement

The authors reported no conflicts of interest.

The *Journal* policy requires editors and reviewers to disclose conflicts of interest and to decline handling or reviewing manuscripts for which they may have a conflict of interest. The editors and reviewers of this article have no conflicts of interest.

## Supplementary Data

Video 1 Video recording demonstrating operative findings and surgical technique. Video available at: https://www.jtcvs.org/article/S2666-2507(26)00365-2/fulltext.

## References

[1] Elgendy I.Y., Conti C.R. (2013). Caseous calcification of the mitral annulus: a review. Clin Cardiol.

[2] Kanemitsu S., Bessho S., Sakamoto S., Yamamoto N., Ito H., Shimpo H. (2020). Calcified amorphous tumor with caseous calcification of mitral annulus in hemodialysis patients. Gen Thorac Cardiovasc Surg.

[3] Fong L.S., McLaughlin A.J., Okiwelu N.L. (2017). Surgical management of caseous calcification of the mitral annulus. Ann Thorac Surg.

[4] Wehman B., Dawood M., Ghoreishi M. (2015). Surgical management of caseous calcification of the mitral annulus. Ann Thorac Surg.

[5] Kanemitsu S., Sakamoto S., Mizumoto T. (2025). Surgical management for caseous calcification of mitral annulus associated with coronary artery disease. Interdiscip Cardiovasc Thorac Surg.

